# Prevalence and correlation of C-shaped root canals of mandibular premolars and molars in Eastern Chinese individuals

**DOI:** 10.1038/s41598-022-24381-5

**Published:** 2022-11-17

**Authors:** Cheng Chen, Tingting Zhu, Huili Wu, Xiao Zhao, Diya Leng, Jingyan Wang, Lianfeng Yang, Daming Wu

**Affiliations:** 1 Department of Endodontics, The Affiliated Stomatological Hospital of Nanjing Medical University, 1 Shanghai Road, Nanjing, 210029 People’s Republic of China; 2 Present Address: Department of Oral & Maxillofacial Imaging, The Affiliated Stomatological Hospital of Nanjing Medical University, 1 Shanghai Road, Nanjing, 210029 People’s Republic of China; 3 Jiangsu Province Key Laboratory of Oral Diseases, Nanjing, China; 4 Jiangsu Province Engineering Research Center of Stomatological Translational Medicine, Nanjing, China

**Keywords:** Anatomy, Diseases, Health care, Medical research

## Abstract

The aim of this study was to investigate the prevalence, correlation, and differences of C-shaped root canals (CSRCs) morphology in permanent mandibular premolars and molars in Eastern Chinese individuals using cone-beam computed tomography (CBCT). A total of 8000 mandibular first premolars (MFPs), mandibular second premolars (MSPs), mandibular first molars (MFMs), and mandibular second molars (MSMs) CBCT images from 1000 patients (692 females and 308 males) were collected. The prevalence, correlation, bilateral/unilateral presence, the morphology of CSRCs, level of canal bifurcation, gender differences, and location of radicular grooves (RGs) were evaluated. The prevalence of CSRCs in MFPs, MSPs, MFMs and MSMs were 10.25%, 0.25%, 0.55% and 47.05%, respectively. The prevalence of CSRCs in MFPs of males was higher than that in females, while the prevalence of CSRCs in MSMs of females was higher than that in males (*P* < 0.05). The bilateral symmetry presence of CSRCs in MSMs was significant but not in MFPs, MSPs, and MFMs. RGs were predominantly found on the mesiolingual (ML) surface of premolars and the lingual surface of molars. There was a high prevalence of CSRCs in MFPs and MSMs in the Eastern Chinese population, but there was no correlation. The prevalence of CSRCs in MFPs and MSMs differ significantly by gender (*P* < 0.05).

## Introduction

Successful root canal treatment requires adequate debridement, shaping, and complement obturation of all root canals in three dimensions (3D), so a thorough knowledge of the anatomy and morphology of the root canals is necessary^1^. There are numerous types of research on root canal morphology, which mainly focus on: root canal morphology in different teeth from different regions and populations^2–4^, and case reports of unique root canal morphology^5–7^. There are currently recognized variances in root canal morphology based on tooth position, race, and gender^2,4,8–11^.

The discovery of C-shaped roots dates back a century and the specific terminology: C-shaped root canals (CSRCs) was first proposed by Cooke and Cox in 1979 when depicting this anatomy in mandibular second molars^12^. Then CSRCs were also identified in mandibular premolars^1^ and maxillary molars^13^. The inability of Hertwig's epithelial root sheath to fuse on the lingual or buccal root surface and the cementum deposition over time may be the leading causes of CSRCs^14^. A fin or web connecting the individual root canals is the primary anatomical feature of CSRCs^15^, and thorough debridement of these anatomical structures is a big challenge^16,17^. In1991, Melton et al. were the first to put forward principles of the CSRCs in molars^18^, and a modified classification based on Melton’s principle was proposed by Fan et al. in 2004, which classified CSRCs into five categories, described the differences between them, and made up for the shortcomings of the initial categorization^15^. The 3D morphology of the CSRCs system was divided into three types: (a) Merging type, (b) symmetrical type, and (c) asymmetrical type^19^.

Many approaches were employed to examine human teeth' internal and exterior structure, such as radiography techniques^20^, cleaning techniques^21^, micro-CT in vitro^22^, and cone-beam computed tomography (CBCT) in vivo^1^. Micro CT produces better image details compared with CBCT^23^. It used extracted teeth as samples for in vitro research, but it is not easy to collect many extracted teeth to match the experiment's requirements, which makes it difficult to understand the relationship of CSRCs with other factors such as sex, location (left or right side), and bilaterality^1^. Furthermore, the reasons why teeth are extracted including complex internal morphologies more difficult to treat or periodontal problems that might be related to the presence of radicular grooves (RGs) caused a misunderstanding of the true prevalence of CSRCs^1,24^. Over the last three decades, the use of CBCT in endodontics has gradually increased and confirmed the values of CBCT on diagnosis, treatment planning, and decision-making^25–27^. CBCT provides a non-invasive 3D confirmatory diagnosis to complement conventional radiography^25^. For endodontists, CBCT is critical in the event of a treatment-related mishap, such as perforation of pulp bottom, root canal perforation, or instrument separation^28^.

The CSRCs in mandibular second molars (MSMs) have been extensively studied in the past. The prevalence of CSRCs in MSMs is much higher in the Asian population than in other races^16,29,30^. Although there are many reports on the prevalence of CSRCs in the Chinese population, large-sample research on the prevalence of CSRCs in mandibular premolars is scarce^1,29^. Furthermore, the prevalence of CSRCs in mandibular first premolars (MFPs) was high in the Chinese population^22,31,32^, while there has been no published research on the relationship between MFPs and MSMs in the Chinese population. There was just one study investigating the relationship between the prevalence of complicated root canals in the mandibular posterior teeth in the Chinese population^32^, which revealed that the prevalence of CSRCs in MFPs will increase when the distolingual root is found in permanent mandibular first molars (MFMs) in the Chinese-Taiwanese population.

The purpose of this study is to investigate the prevalence, correlation, and differences of CSRCs morphology in permanent mandibular premolars and molars in Eastern Chinese individuals utilizing CBCT.

## Methods and materials

### Image acquisition

CBCT images with MFPs, mandibular second premolars (MSPs), MFMs, and MSMs with completely developed roots were collected. The presence of unclear images, posts or crowns, periapical lesions, and endodontic treatments were excluded. A total of 8000 mandibular premolars and molars CBCT images from 1000 patients (692 females and 308 males) were collected (Table 1). These 1000 patients are all of Han nationality.

**Table 1:** Distribution of males and females in different age groups.

Age	Males	Females	Sum
≤ 20	88 (40.55%)	129 (59.45%)	217
21–30	156 (29.83%)	367 (70.17%)	523
31–40	46 (23.71%)	148 (76.29%)	194
41–50	15 (30.61%)	34 (69.39%)	49
≥ 51	3 (17.65%)	14 (82.35%)	17
Sum	308 (30.80%)	692 (69.20%)	1000
*P* value	0.003		

There was a significant difference in the proportion of gender among age groups.

Sum: Summation.

All images were obtained from a CBCT database from the Department of Oral & Maxillofacial Imaging, the Affiliated Stomatological Hospital of Nanjing Medical University. Images were gathered from the patients who required CBCT imaging as part of their dental evaluation for orthodontics, implants, trauma, temporomandibular joint diseases, and other reasons between January and October 2021. The Ethical Committee Department of the Affiliated Stomatological Hospital of Nanjing Medical University approval (PJ2021-116-001) was obtained, and as recommended by the Research and Ethics Committee, formal consent is not required.

The CBCT images were obtained using a CBCT scanner (NewTom 5G, QR s.r.l., Italy) at 110 kV and 4–14 mA with an exposure time of 3–5 s and FOV (Field of view) of 16 × 18 cm or 15 × 12 cm. The voxel size of the images and axial thickness was 0.30 mm. According to the manufacturer's recommended protocol, an experienced radiologist performed the acquisition process.

### Image evaluation

Two endodontists independently evaluated the images twice using NNT 10.0.0 software (QR s.r.l., Verona, Italy). A radiologist with experience in endodontics was invited to perform a third evaluation and reach a final consensus when existing disagreements. Their measurements were calibrated before the experiment by reading 20 CBCT images of CSRCs in premolars and molars chosen to ensure the accuracy of the results. Cohen's kappa statistical analysis was used to assess the intra- examiner and inter-examiner reliability.

The cross-sectional configurations were analyzed to determine the frequency of CSRCs at different axial levels in premolars and molars: “A,” the coronal-third point (1/3 the distance between the orifice and the anatomical apex), “B,” the middle of the roots (mid-point from orifice to apex distance-wise); “C,” the apical-third point (junction between the middle and apical thirds of the root distance-wise); “D,” 2 mm from the anatomical apex. The mandibular molars were defined as CSRCs when exhibiting all the characteristics: fused roots, a longitudinal groove on the root’s lingual or buccal surface, and at least one cross-section of the canal belonging to the C1, C2, or C3 configuration according to Fan’s classification^15^:

- C1: the shape was a continuous “C” with no separation or division.
- C2: the canal shape resembled a semicolon resulting from a discontinuation in the “C” outline.
- C3: two separate round, oval, or flat canals.
- C4: only one round or oval canal in that cross-section.
- C5: no canal lumen could be observed.

All mandibular premolars were defined as CSRCs when exhibiting all the characteristics: fused roots, a radicular groove on the root’s surface, and at least one cross-section of the canal belonging to the C1 or C2 configuration^33^.

**Figure 1:**
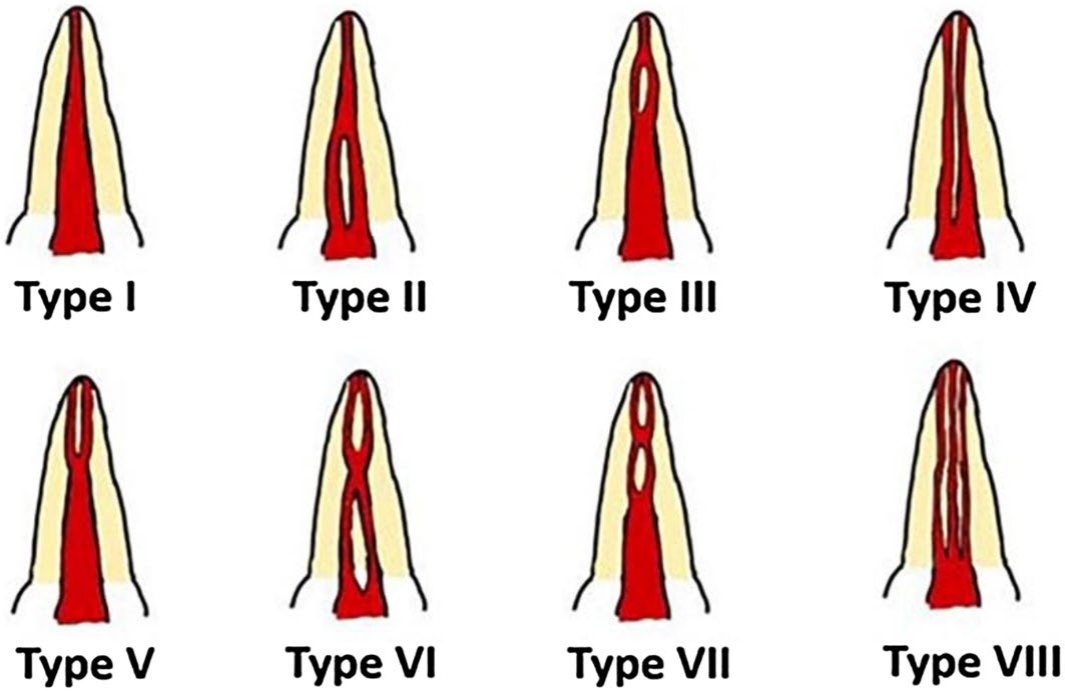
Classification of root canal configuration. Type I; a single canal extends from the pulp chamber to the apex. Type II; two separate canals leave the pulp chamber and join short of the apex to form one canal. Type III; one canal leaves the pulp chamber, divides into two within the root, and then merges to exit as one canal. Type IV; two separate and distinct canals extend from the pulp chamber to the apex. Type V; one canal leaves the pulp chamber and divides short of the apex into two separate and distinct canals with separate apical foramina. Type VI; two separate canals leave the pulp chamber, merge in the body of the root, and re-divide short of the apex to exit as two distinct canals. Type VII; one canal leaves the pulp chamber, divides and then rejoins within the body of the root, and finally re-divides into two distinct canals short of the apex. Type VIII; three separate and distinct canals extend from the pulp chamber to the apex.

Age, gender, tooth position (left or right side), morphology of CSRCs, Vertucci root canal classification of premolars presenting CSRCs^34^ (Fig. 1), and location of RGs were recorded.

### Statistical analysis

The prevalence of CSRCs was expressed with a 95% confidence interval (CI). The Chi-square test and Fisher’s exact test were used to analyze the correlation between the prevalence of CSRCs and RGs in different tooth positions, age groups, and sex difference by SPSS 25.0 software (SPSS Inc, Chicago, IL, USA). Differences were statistically significant when *P* was < 0.05.

## Results

CBCT evaluations revealed no intra-observer variance for either of the observers (*P* > 0.05). Intra-examiner and inter-examiner agreements had kappa values of 0.875 to 0.902, respectively.

### Mandibular first premolars

The prevalence of CSRCs in the MFPs was 10.25% (205/2000; 95% CI 8.9–11.6%), of which were 16.23% (100/616; 95%CI 13.3–19.2%) in males and 7.59% (105/1384; 95%CI 6.2–9.0%) in females. The prevalence of RGs in the MFPs was 21.10% (422/2000; 95%CI 19.3–22.9%), of which were 26.95% (166/616; 95%CI 23.4–30.5%) in males and 18.50% (256/1384; 95%CI 16.4–20.5%) in females. The prevalence of CSRCs and RGs in males was higher than in females (*P* < 0.05). There was no statistical difference in the prevalence of CSRCs and RGs in different tooth positions (Table 2).

**Table 2:** Distribution of CSRCs and RGs in different tooth positions and sex.

		CSRCs		*P* value	RGs		*P* value
		Males (308)	Females (692)		Males (308)	Females (692)	
Left	MFPs^a^	49 (15.90%)	53 (7.65%)	< 0.001	81 (26.29%)	130 (18.78%)	0.007
	MSPs	1 (0.32%)	2 (0.28%)	1	2 (0.64%)	4 (0.57%)	1
	MFMs	1 (0.32%)	3 (0.43%)	1	1 (0.32%)	3 (0.43%)	1
	MSMs^a^	99 (32.14%)	369 (53.32%)	< 0.001	99 (32.14%)	369 (53.32%)	< 0.001
Right	MFPs^a^	51 (16.55%)	52 (7.51%)	< 0.001	85 (27.59%)	126 (18.20%)	0.001
	MSPs	0	2 (0.28%)	1	2 (0.64%)	0	0.095
	MFMs	1 (0.32%)	6 (0.86%)	0.683	1 (0.32%)	6 (0.86%)	0.683
	MSMs^a^	110 (35.71%)	363 (52.45%)	< 0.001	110 (35.71%)	363 (52.45%)	< 0.001

CSRCs: C-shaped root canals, RGs: radicular grooves, MFPs: mandibular first premolars, MSPs: mandibular second premolars, MFMs: mandibular first molars, MSMs: mandibular second molars.

^a^The prevalence of CSRCs in MFPs of males was higher than that in females, while the prevalence of CSRCs in MSMs of females was higher than that in males.

RGs were mainly located on the mesiolingual (ML) surface of the root, and there was no significant difference in the prevalence of CSRCs in different types of RGs (*P* > 0.05). Root canal bifurcations were primarily located in the middle third, and the prevalence of CSRCs was significantly lower in the non-bifurcation group than that in the other group (*P* < 0.05) (Table 3). The canal shape in CSRCs could vary along the length of the root. C4 (77.07%, 158/205) prevailed on the axial of A. C3 (52.20%, 107/205) dominated the axial of B. C2 (54.63%, 112/205) played the most crucial role on the axis of C, followed by C3 (25.37%, 52/205). C3 (60.00%, 123/205) dominated the axial of D. Detailed morphology of different axials in CSRCs was presented in (Table 4).

**Table 3:** The prevalence of CSRCs in different types of RGs and canal bifurcations.

		MFPs	MSPs
Types of RGs (CSRCs)	Mesiolingual	368 (175)	2 (1)
	Mesial	1 (0)	
	Distal		1 (1)
	Buccal	3 (3)	
	Lingual	37 (20)	5 (3)
	Mesiolingual and Buccal	12 (6)	
	Buccal and Lingual	1 (1)	
	*P* value	0.338	1
Types of canal bifurcations (CSRCs)	Coronal	79 (47)	1 (1)
	Middle	214 (122)	2 (2)
	Apical	31 (13)	
	Non	98 (23)^a^	5 (2)
	*P* value	< 0.001	0.643

CSRCs: C-shaped root canals, RGs: radicular grooves, MFPs: mandibular first premolars, MSPs: mandibular second premolars.

^a^The prevalence of CSRCs in Non-group was significantly lower than that in the other group.

**Table 4:** Configurations of different axials in mandibular premolars and molars.

	MFPs				MSPs				MFMs				MSMs			
	A	B	C	D	A	B	C	D	A	B	C	D	A	B	C	D
C1	0	37	25	6	0	3	0	0	4	1	1	0	518	230	264	56
C2	0	48	112	0	0	1	2	0	4	4	5	1	338	501	402	222
C3	47	107	52	123	0	1	1	1	1	6	5	8	32	205	178	152
C4	158	13	15	56	5	0	2	3	2	0	0	2	53	4	79	479
C5	0	0	1	20	0	0	0	1	0	0	0	0	0	1	18	32

MFPs: mandibular first premolars, MSPs: mandibular second premolars, MFMs: mandibular first molars, MSMs: mandibular second molars.

**Figure 2:**
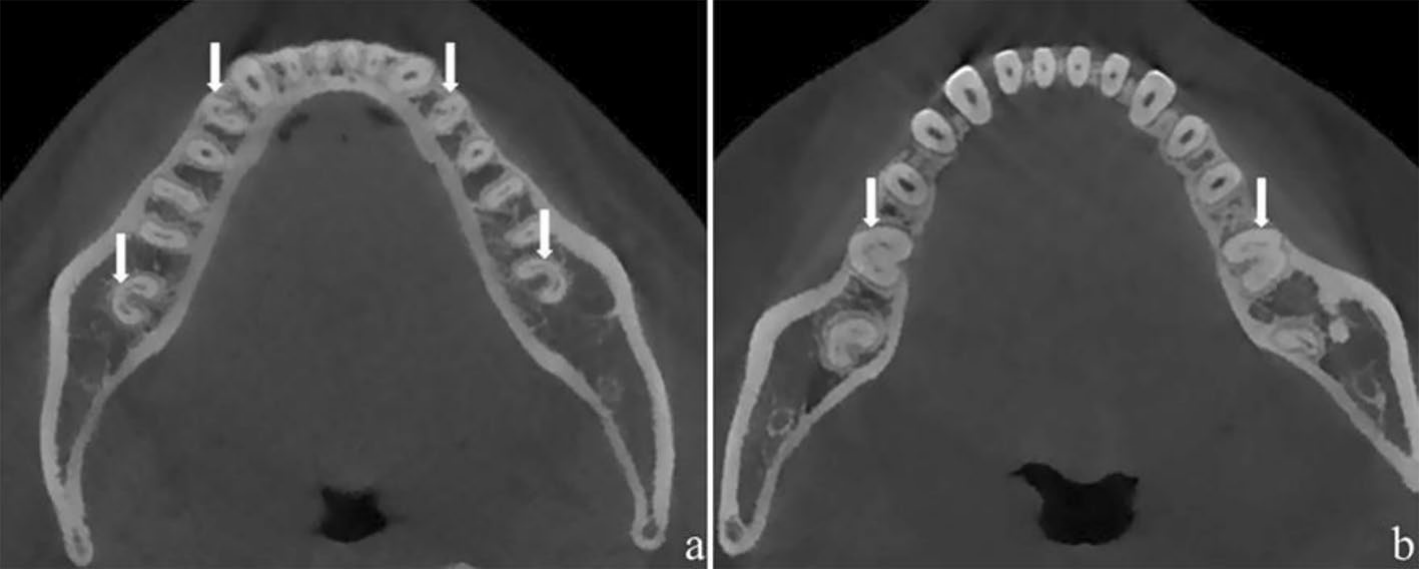
(**a**) Bil-CSRCs in MFPs and MSMs, (**b**) Bil-CSRCs in MFMs.

According to Vertucci classification, Type V (72.68%, 149/205) was the primary morphology of MFPs presenting CSRCs, followed by type III (15.61%, 32/205), type I (10.24%, 21/205), and type VII (0.49%, 1/205). The bilateral symmetry presence of CSRCs was not significant in MFPs (Fig. 2a; Table 5). The prevalence of CSRCs in the contralateral tooth increased when presented on one side. The Chi-square test revealed inter-group disparities in the prevalence of CSRCs across age groups (Table 6).

**Table 5:** The symmetry of CSRCs (RGs) in different tooth positions.

	MFPs	MSPs	MFMs	MSMs
Bilateral	55 (162)	0 (0)	2 (2)	389 (389)
Unilateral	95 (98)	5 (8)	7 (7)	163 (163)
Non	850 (740)	995 (992)	991 (991)	448 (448)

CSRCs: C-shaped root canals, RGs: radicular grooves, MFPs: mandibular first premolars, MSPs: mandibular second premolars, MFMs: mandibular first molars, MSMs: mandibular second molars.

**Table 6:** Prevalence of CSRCs in different tooth positions among different age groups.

Age	MFPs*	MSPs	MFMs	MSMs
≤ 20 (434)	67_a_ (15.44%)	1 (0.23%)	2 (0.46%)	214 (49.31%)
21–30 (1046)	109_b_ (10.42%)	2 (0.19%)	6 (0.57%)	500 (47.80%)
31–40 (388)	17_c_ (4.38%)	1 (0.26%)	0	179 (46.13%)
41–50 (98)	8_a,b,c_ (8.16%)	1 (1.02%)	0	38 (38.78%)
≥ 51 (34)	4_a,b,c_ (11.76%)	0	1 (2.94%)	10 (29.41%)
*P* value	< 0.001	0.423	0.158	0.085

CSRCs: C-shaped root canals, MFPs: mandibular first premolars, MSPs: mandibular second premolars, MFMs: mandibular first molars, MSMs: mandibular second molars.

*The Z-test revealed there was a significant difference in the prevalence of CSRCs in MFPs among age groups (each subscript letter denotes a subset of age categories whose column proportions do not differ significantly from each other at the 0.05 level).

### Mandibular second premolars

The prevalence of CSRCs in the MSPs was 0.25% (5/2000; 95%CI 0–0.5%), of which were 0.49% (3/616; 95%CI 0.1–1%) in males and 0.14% (2/1384; 95%CI 0.1–0.3%) in females. The prevalence of RGs in the MSPs was 0.40% (8/2000; 95%CI 0.1–0.7%), of which were 0.65% (4/616; 95%CI 0–1.3%) in males and 0.29% (4/1384; 95%CI 0–0.6%) in females (Table 2). There was no statistical difference between males and females in the prevalence of CSRCs and RGs in MSPs (*P* > 0.05). There was no statistical difference in the prevalence of CSRCs and RGs in different positions (*P* > 0.05).

RGs were mainly located on the lingual surface of the root, and there was no significant difference in the prevalence of CSRCs in different types of RGs (*P* > 0.05). Root canal bifurcations were primarily located in the middle third, and there was no difference in the prevalence of CSRCs in different canal bifurcations (Table 3). Detailed morphology of different axials in CSRCs was presented in (Table 4). According to Vertucci’s criteria, type I (40%, 2/5) and type III (40%, 2/5) were the primary morphologies in MSPs presenting CSRCs, followed by type V (20%, 1/5). Regarding the symmetry presence of CSRCs and RGs in MSPs, no one presented this condition in any teeth (Table 5). The Chi-square test revealed no difference in the prevalence of CSRCs across age groups (Table 6).

**Figure 3:**
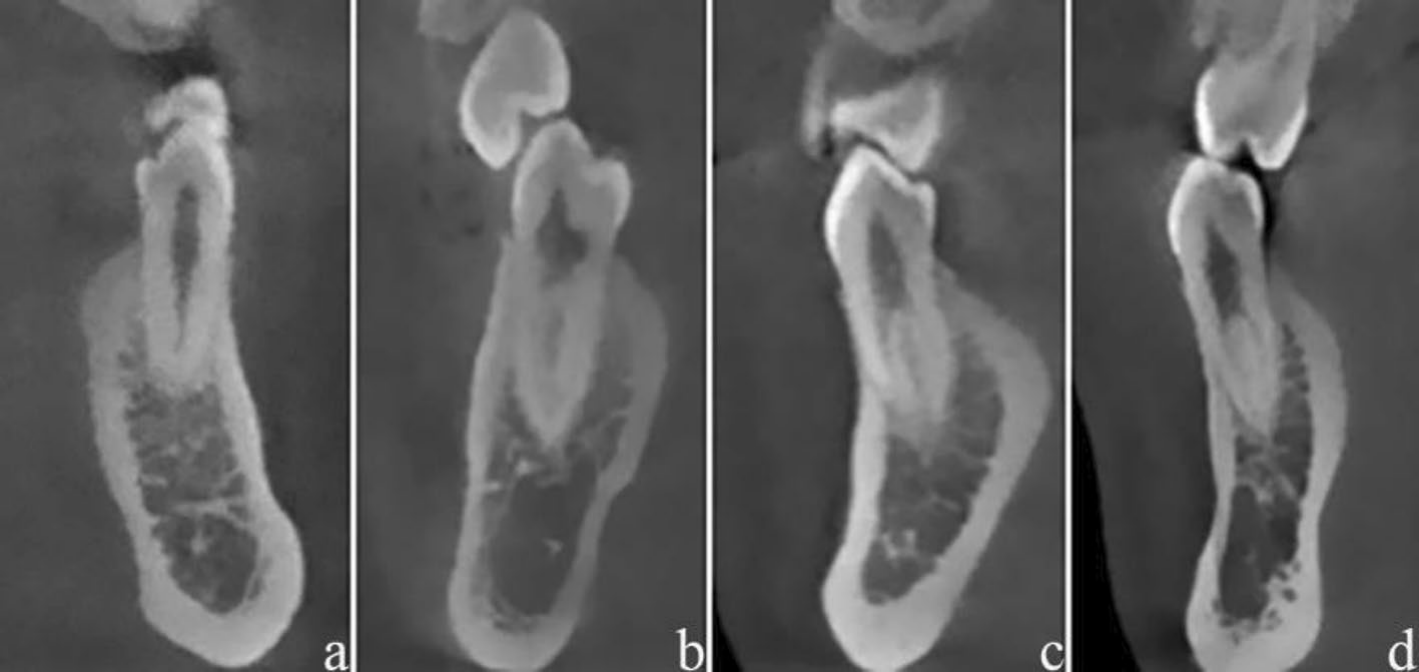
Several examples of root canal system configurations in mandibular premolars according to Vertucci classification (**a**: I; **b**: III; **c**: V; **d**: VII).

**Figure 4:**
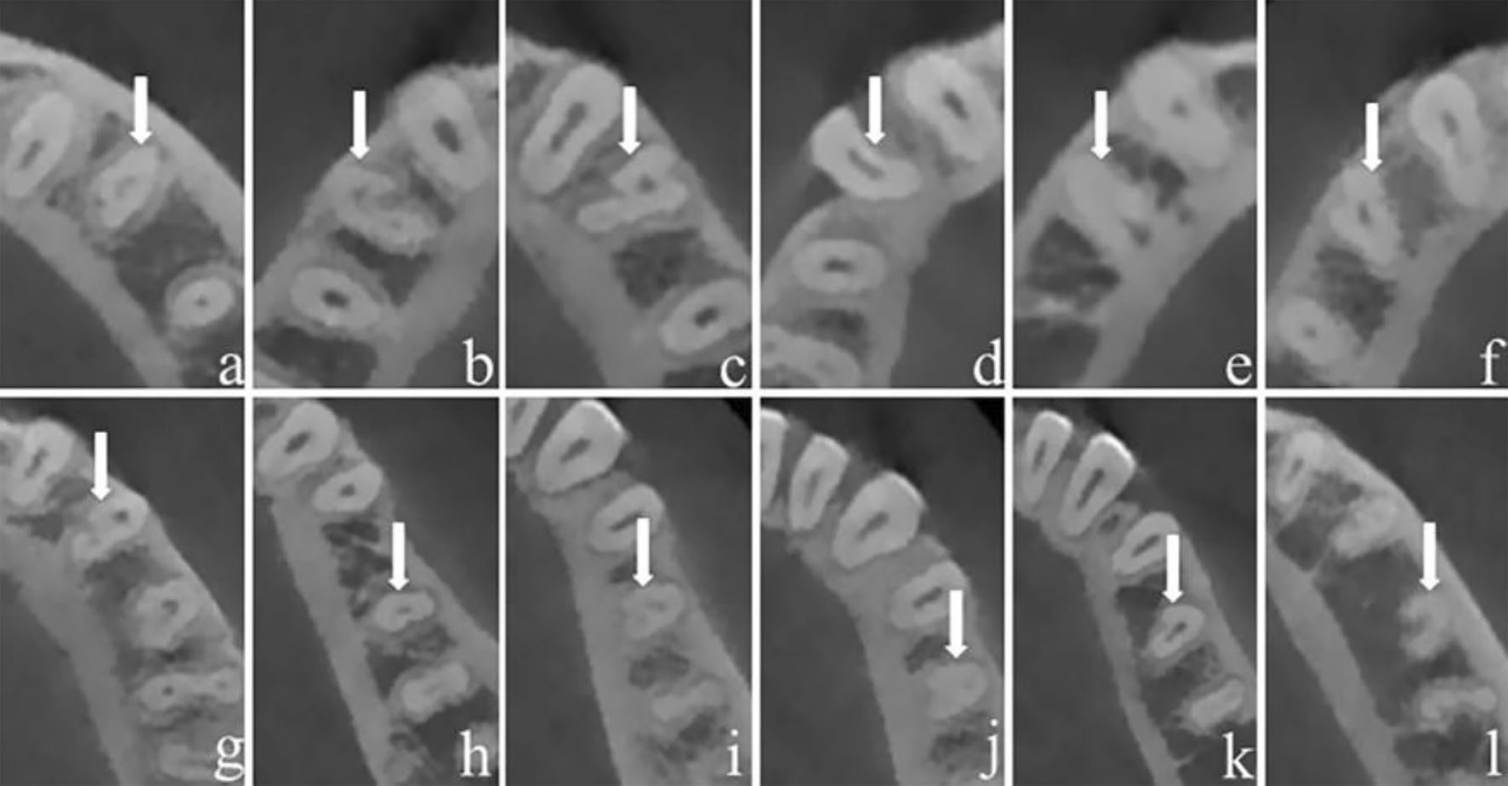
Different types of CSRCs and RGs in MFPs (**a**, C1, Lingual; **b**, C2, ML; **c**, C3, ML and Buccal; **d**, C4, Mesial; **e**, C5, Lingual; **f**, C4, Buccal; **g**, C3, ML;) and MSPs (**h**, C1, Distal; **i**, C2, Lingual; **j**, C3, Lingual; **k**, C4, ML; **l**, C5, Lingual).

Several examples of root canal system configurations and different CSRCs and RGs in mandibular premolars were shown in Figs. 3 and 4.

### Mandibular first molars

The prevalence of CSRCs in the MFMs was 0.55% (11/2000; 95%CI 0.2–0.9%), of which were 0.32% (2/616; 95%CI 0.1–0.8%) in males and 0.65% (9/1384; 95%CI 0.2–1.1%) in females (Table 2). There was no difference in the prevalence of CSRCs in gender and dental position (*P* > 0.05).

RGs were mainly located on the root’s lingual surface (54.55%, 6/11), followed by buccolingual (45.45%, 5/11). A detailed analysis of different axials in CSRCs was presented in (Table 4). Regarding the symmetry presence (Table 5), CSRCs in MFMs occurred bilaterally in 0.20% (2/1000) of patients (Fig. 2b), unilaterally in 0.70% (7/1000), or on neither side in 99.10% (991/1000). The bilateral symmetry presence of CSRCs was not significant in MFMs (*P* > 0.05). The Chi-square test revealed no difference in prevalence across age groups (*P* > 0.05) (Table 6).

### Mandibular second molars

Of 2000 MSMs evaluated, 941(47.05%;95%CI 44.9–49.2%) were classified as CSRCs, with 732 in females (52.89%; 95%CI 50.3–55.5%) and 209 in males (33.92%; 95%CI 30.2–37.7%) (Table 2). The Chi-square test showed a higher chance of CSRCs occurring in females than males (*P* < 0.05). There was no difference in the prevalence of CSRCs in dental positions (*P* > 0.05).

Most of the grooves were located on the lingual surface (64.72%, 609/941) of the root, followed by the buccolingual (35.07%, 330/941) and the buccal (0.21%, 2/941). C1 (55.04%, 518/941) and C2 (35.92%, 338/941) prevailed on the axial of A. C2 (53.24%, 501/941) dominated the axial of B. C2 (42.72%, 402/941) and C1 (28.06%, 264/941) played the most critical roles on the axis of C, followed by C3 (18.92%, 178/941).C4 (50.90%, 479/941) dominated the axial of D (Table 4).

Regarding symmetry (Table 5), CSRCs in MSMs occurred bilaterally in 38.9% (389/1000) of patients, unilaterally in 16.3% (163/1000), or on neither side in 44.80% (448/1000). In patients presenting CSRCs, 70.47% (389/552) had a bilateral condition (Fig. 2a), and 29.52% (163/552) had a unilateral condition. The bilateral symmetry presence of CSRCs in MSMs was significant. The Chi-square test revealed no inter-group disparities in the prevalence of CSRCs across age groups (*P* > 0.05) (Table 6).

**Figure 5:**
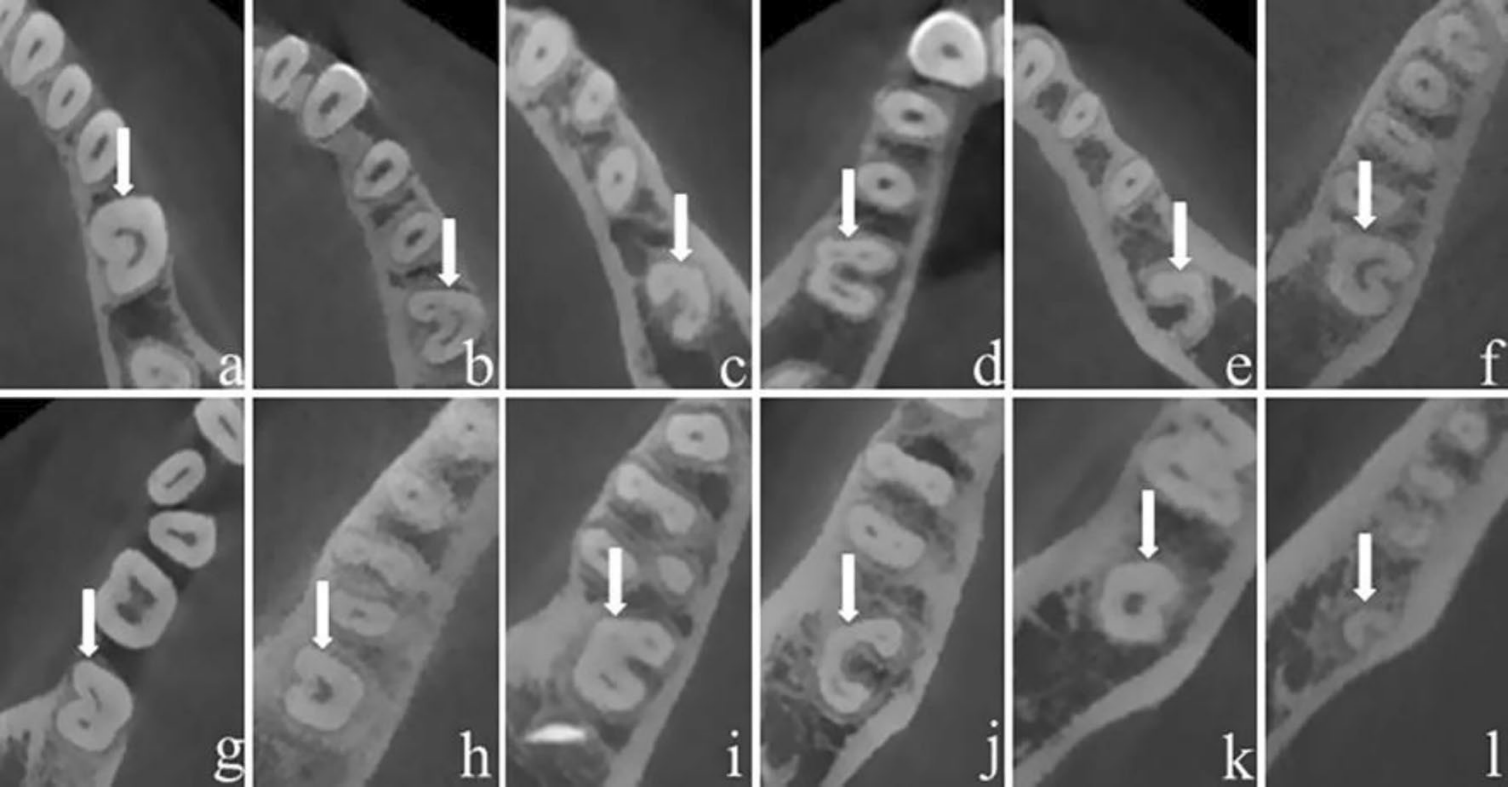
Different types of CSRCs and RGs in MFMs (**a**, C1, Lingual; **b**, C2, Lingual; **c**, C3, Lingual; **d**, C3, Buccolingual; **e**, C5, Lingual;) and MSMs (**f**, C1, Lingual; **g**, C2, Buccolingual; **h**, C1, Buccal; **i**, C3, Lingual; **j**, C3, Lingual; **k**, C4, Lingual; **l**, C5, Lingual).

Different CSRCs and RGs in mandibular molars were presented in (Fig. 5).

### Ethics approval and consent to participate

This study was performed in line with the principles of the Declaration of Helsinki. Approval was granted by the Ethical Committee Department of the Affiliated Stomatological Hospital of Nanjing Medical University (PJ2021-116-001). Through this approval, the research team had the required administrative permissions to access the data used in this research. For this type of retrospective study, and as recommended by the Research and Ethics Committee, formal consent is not required.

## Discussion

The prevalence of CSRCs in mandibular posterior teeth varies between countries and teeth, 1%^21^ to 28.94%^35^ in MFPs, 0%^36^ to 7.14%^35^ in MSPs, 0%^37–41^ to 24.01%^42^ in MFMs, and 1.9%^43^ to 48.7%^41^ in MSMs. Surprisingly, there was also a large variation in the prevalence of CSRCs in the same tooth position within the same country, such as Brazil^42,44,45^, Thailand^46,47^, and China^22,31,32,48–50^. Both China and Brazil are multi-ethnic countries, which may be one reason for the different prevalence rates, in addition to differences in sample size, methods, and definition of CSRCs. Disagreement over the definition of CSRCs exists mainly among premolars, one study adopted C1 as standard^36^, some studies considered C1 and C2 as standard^1,33,46,51^, and others included C3 or C4^31,35,47,49,52,53^.

The optimal sample size means that there are enough patients included in the study to find a statistically significant or clinically worthwhile effect if there is one. More patients than necessary are not recommended to save research time and money^54^. A study examining the prevalence of CSRCs in MSMs from different regions of the world used a sample size of 400 teeth per region, which was chosen because the final proportion of a pilot study stabilized at 400, and did not change significantly as the measure increased^16^. When using CBCT images to study the prevalence of anatomical structures, fewer than half of the studies selected the correct sample size, and the JBI assessment methodology was recommended to determine sample size^55^.

In this study, the prevalence of CSRCs in MFPs of males was higher than that in females (*P* < 0.05). This study was the first to find gender differences in the prevalence of CSRCs in Chinese MFPs. This finding was consistent with other studies^1,46^ but contradicted other studies^30,35,47^. Two studies^46,47^ investigating the Thai population reached different conclusions on whether there is a gender difference in the occurrence of CSRCs in premolars. The main reason for this difference may be the definition of CSRCs, except sample size. CSRCs in MSMs were more common in women, consistent with other recent studies worldwide^16,42,45,56–59^. There are also reports that there is no gender difference in the prevalence of CSRCs^60–62^. The main reason may be racial differences and sample size. No significant differences were found in gender concerning the presence of CSRCs in MSPs and MFMs in this research. Only one study^42^ has reported a significantly higher prevalence of CSRCs in women than men in first molars. The main reason for this difference may be racial differences.

We did not find an effect of the left or right side on the prevalence of CSRCs in MFPs, MSPs, MFMs, and MSMs, which was consistent with the conclusions of other studies^1,57–59,61,62^. We found no studies reporting significant differences between different tooth positions. In this study, the bilateral symmetry presence of CSRCs was not significant in MFPs, MSPs, and MFMs, while patients with bilateral presence of the CSRCs in MSMs were more common. This result was consistent with some studies^1,16,42,47,51,52,57,58,62^ but contradicted other studies^32,47,53,56,61^. We did not find patients with bilateral CSRCs in MSPs. So far, only one study claimed that C-shaped root canal symmetry was significant in MFMs^42^. In the other two studies^56,61^, only one patient presented CSRCs in MFMs, leading to an underestimation of whether symmetry occurred. The main reason for these differences may be the low prevalence of CSRCs, different methods adopted, sample size, the definition of CSRCs, and ethnic differences.

RGs in MFPs and MSPs were mainly located on the ML surface of the root. This finding was consistent with earlier studies^1,22,30,35,46,51–53,63^. Most of the RGs in MFMs and MSMs were located on the lingual surface of the root. This finding was consistent with some studies^30,57,61,62^**. **Root canal bifurcations in MFPs and MSPs were mainly located in the middle of the root, consistent with other studies^46,47,51^.

In this research, type V was the main configuration in MFPs presenting CSRCs, which was consistent with other studies^1,32,33,47,51^. Type III (40%, 2/5) and type I (40%, 2/5) were the primary morphologies of MSPs presenting CSRCs. Since the prevalence of CSRCs in MSPs was very low, this result is not very informative.

The most common root canal configuration in MFPs presenting CSRCs was C3. This result was consistent with Wu et al.^32^ and Fan et al.^22^ but contradicted Martins et al.^1^. The main reason for this difference was the diverse axials selected, apart from race and sample size disparities. C1 was mainly found in the middle of the root, and this result was consistent with Fan et al.^22^ and Martins et al.^1^. C4 was mainly located in the coronal third, and C5 was found primarily in the apical third, like the findings in other studies^1,22^. In this research, C1 was the primary canal morphology at the coronal third in MSMs showing CSRCs, consistent with Kim et al.^58^ and Vaz de Azevedo et al.^42^. C1, C2, and C3 prevailed in the middle third. C2 and C4 were the main configurations in the apical third. This finding was also consistent with Alfawaz et al.^56^ but contradicted Kim et al.^58^. Ethnic differences may be a prominent cause. Morphological analysis of large samples of MSPs and MFMs is still lacking.

The Chi-square test revealed inter-group disparities in the prevalence of CSRCs in MFPs across age groups. So far, few reports about the prevalence of CSRCs in MFPs showed significant differences between age groups. Only one study reported that the higher prevalence of CSRCs in molars was found among the 45–54 years group with 11.1%, while the lowest rate was found in the 65–74 years group with a prevalence of 5.3%^59^. There are two reasons to explain the disparities. Firstly, there was a significant difference in the proportion of gender among age groups. Secondly, the prevalence of CSRCs in males of first premolars is significantly higher than that in females.

In this study, the correlations between the prevalence of the CSRCs in mandibular premolars and molars were investigated. The results showed that the prevalence of CSRCs in MFPs was significantly higher than that of MSPs (*P* < 0.05), and the prevalence of CSRCs in MSMs was considerably higher than that of MFMs (*P* < 0.05). There was no relationship between CSRCs in MFPs and MSMs, consistent with MH Mashyakhy’s research^30^, suggesting to clinicians that the occurrence of CSRCs in MSMs does not imply an increased chance of CSRCs occurring in the ipsilateral MFPs. In this study, no patient had a C‑shaped system in MFPs, MSPs, MFMs, and MSMs simultaneously.

One limitation of our work was that it was a retrospective study. We cannot control for characteristics such as FOV and voxel size. A voxel size of 0.250 mm or 0.200 mm was effective in recognizing C-shaped morphologies in earlier research^1,16,30,36,41,42,45,47,56,59^, and 0.3 mm voxel has only been used in few studies^43,64^. CBCT must be justified, like any other radiographic examination, and the potential benefits must balance the risk of ionizing radiation exposure^25^. Each examination should be tailored to the specific patient and their diagnostic requirements^26^. The principle of ALARA (as low as reasonably achievable) must be followed. Most CBCT images investigated in this study came from CBCT images taken for temporomandibular joint disease diagnosis and standard pre-orthodontic examinations. The 0.3 mm voxel can meet the needs of orthodontic and temporomandibular joint surgeons.

This study was the first to investigate the relationship and differences between CSRCs in different mandibular tooth positions in the Eastern Chinese population and find gender differences in the prevalence of CSRCs in Chinese MFPs. Large-sample research on the prevalence of CSRCs in mandibular premolars is scarce, this research provided the prevalence of CSRCs in MFPs in the Eastern Chinese population with large sample size. Interestingly, we found no significant difference in the prevalence of CSRCs in different types of RGs, and the prevalence of CSRCs was significantly lower in the non-bifurcation group. There have been no relevant studies reported so far. According to previous assumptions, cementum deposition may lead to C-shaped roots^14^. Compare the CBCT images of the same patient in young and old age stages to confirm the validity of this hypothesis, which will be an exciting direction for future research, and whether there is family inheritance in the occurrence of CSRCs deserves to investigate. CSRCs in the maxillary second molar were not unusual^65^. Whether there is a connection between the upper and lower jaws for the occurrence of C-shaped root canals in the dentition may be a direction for future research.

## Conclusions

There was a high prevalence of CSRCs in MFPs and MSMs in the Eastern Chinese population, but there was no correlation. The prevalence of CSRCs in MFPs and MSMs differ significantly by gender (*P* < 0.05). The bilateral symmetry presence of CSRCs in MSMs was significant but not in MFPs, MSPs, and MFMs. Root canal bifurcations of premolars presenting CSRCs were primarily located in the middle third. The prevalence of CSRCs in the contralateral tooth increased when presented on one side. Clinicians should pay attention to this phenomenon. In the process of root canal treatment of teeth with CSRCs, CBCT should be taken if necessary, and the treatment should be carried out with the aid of a microscope.

## Data Availability

The datasets used and analyzed during the current study are available from the corresponding author on reasonable request.

## Abbreviations

CSRCs: C-shaped root canals
RGs: Radicular grooves
CBCT: Cone beam computed tomography
MFPs: Mandibular first premolars
MSPs: Mandibular second premolars
MFMs: Mandibular first molars
MSMs: Mandibular second molars
3D: Three dimension
SPSS: Statistical Package for the Social Sciences
FOV: Field of view
CI: Confidence interval
ML: Mesiolingual
